# Adjudin protects against cerebral ischemia reperfusion injury by inhibition of neuroinflammation and blood-brain barrier disruption

**DOI:** 10.1186/1742-2094-11-107

**Published:** 2014-06-14

**Authors:** Tengyuan Liu, Tingting Zhang, Hemei Yu, Hailian Shen, Weiliang Xia

**Affiliations:** 1 State Key Laboratory of Oncogenes and Related Genes, Renji-Med X Clinical Stem Cell Research Center, Ren Ji Hospital, School of Medicine, Shanghai Jiao Tong University, 1630 Dongfang Road, Shanghai 200127, China; 2 School of Biomedical Engineering & Med-X Research Institute, Shanghai Jiao Tong University, 1954 Huashan Road, Shanghai 200030, China

**Keywords:** ischemia/reperfusion, adjudin, neuroinflammation, blood-brain barrier, MMP-9

## Abstract

Neuroinflammation mediated by activation of microglia and interruption of the blood-brain barrier (BBB) is an important factor that contributes to neuron death and infarct area diffusion in ischemia reperfusion injury. Finding novel molecules to regulate neuroinflammation is of significant clinical value. We have previously shown that adjudin, a small molecule compound known to possess antispermatogenic function, attenuates microglia activation by suppression of the NF-κB pathway. In this study we continued to explore whether adjudin could be neuroprotective by using the transient middle cerebral artery occlusion (tMCAO) model. Adjudin treatment after reperfusion significantly decreased the infarction volume and neuroscore compared to the vehicle group. Staining of CD11b showed that adjudin markedly inhibited microglial activation in both the cortex and the striatum, accompanied by a reduction in the expression and release of cytokines TNF-α, IL-1β and IL-6. Concomitantly, adjudin noticeably prevented BBB disruption after ischemia and reperfusion, as indicated by the reduction of IgG detection in the brain cortex and striatum versus the vehicle group. This finding was also corroborated by immunofluorescence staining and immunoblotting of tight junction-related proteins ZO-1, JAM-A and Occludin, where the reduction of these proteins could be attenuated by adjudin treatment. Moreover, adjudin obviously inhibited the elevated MMP-9 activity after stroke. Together these data demonstrate that adjudin protects against cerebral ischemia reperfusion injury, and we present an effective neuroinflammation modulator with clinical potential.

## Introduction

Stroke has become one of the major causes of death in China and is the leading cause of permanent disability and mortality worldwide [1]. Ischemia/reperfusion-induced cerebral injury not only causes great pain to the patient, but also brings enormous burden to the family as well as to society as a whole. However, effective therapy is yet to be discovered. Among the various underlying mechanisms of stroke, inflammation plays an important role in the pathogenesis of ischemia/reperfusion-induced cerebral injury, although the pathway involved is still largely unrevealed [2]. Inflammatory response could be triggered within a few hours after reperfusion and reaches its peak in the following 3 to 5 days [3]. Dynamic balance between the pro-inflammation and anti-inflammation reactions is disrupted because of the activation of microglia, a resident macrophage-like cell in the brain, and the infiltration of macrophages from the blood after the blood brain barrier (BBB) collapses [4-6]. Over-activated microglia and the disrupted BBB further exacerbate the inflammation and contribute to the spreading of the infarction area [7-9]. Finding an effective anti-inflammation drug that is administered at an appropriate window to regulate microglia activation and protect the BBB could be an efficient approach to protect the brain against stroke-induced damage. In fact, many studies have indicated that inhibition of inflammation after stroke with anti-inflammation drugs could decrease the infarct area and the neuroscore [1]. Adjudin (1-(2,4-dichlorobenzyl)-1H-indazole-3-carbohydrazide), formerly called AF-2364, is a reversible antispermatogenic compound and a potential male contraceptive that can disrupt adherens junctions between germ cells and supporting cells, leading to the exfoliation of germ cells from the seminiferous epithelium [10,11]. It has been reported that a number of indazole derivatives that are used as non-steroidal anti-inflammatory drugs (NSAID) could suppress the production of nitric oxide (NO) and the release of cytokines and chemokines [12]. In our previous work, we demonstrated that adjudin could attenuate lipopolysaccharide (LPS)-induced BV2 activation by suppression of the NF-κB pathway and could reduce microglial activation in permanent middle cerebral artery occlusion (pMCAO) mouse model [13]. However, in that study, a number of questions were not answered [13]. First, since adjudin was administered two hours before ischemia, post-treatment of adjudin that is more clinically relevant needs be tested. Second, brain edema could be reduced after adjudin pre-treatment, but the BBB function was not examined. These issues warrant further investigation.

In this work, we try to explore if adjudin could attenuate microglial activation, help protect BBB integrity and improve behavioral score after ischemia/reperfusion by using the transient middle cerebral artery occlusion (tMCAO) mouse model, which is more relevant to clinical stroke scenarios.

## Materials and methods

### Reagents and animals

DMSO was purchased from Sigma Aldrich (St. Louis, MO, USA). Adjudin was provided by Dr. C Yan Cheng of the Mary M. Wohlford Laboratory, Population Council, New York. Mice were purchased from Shanghai SLAC Laboratory Animal Corporation (Shanghai, China).

### Surgical procedures

Animal surgical procedures and experimental protocols were reviewed and approved by the Institutional Animal Care and Use Committee (IACUC) of Shanghai Jiao Tong University School of Biomedical Engineering. Adult male ICR mice weighing 25 to 30 g were used in the study. Mice were randomly assigned to the adjudin-treated group, the DMSO-treated group or the sham group. The surgical procedure of tMCAO was described previously [14]. Briefly, mice were anesthetized with ketamine (100 mg/kg) and xylazine (10 mg/kg) intraperitoneally. Mice were placed supinely on a heating pad (RWD Life Science, Shenzhen, China), which maintains body temperature at 37.0 ± 0.5°C. The left common carotid artery (CCA), the external carotid artery (ECA) and the internal carotid artery (ICA) were isolated. 6-0 suture (Dermalon, 1741-11, Covidien, OH, USA) coated with silicone was introduced into the ECA stump and advanced from the ICA to the opening of the middle cerebral artery (MCA) until a slight resistance was felt. At this moment, the tip of the suture was located in the anterior cerebral artery (ACA). All procedures were performed under an operating microscope (Leica, Wetzlar, Germany). The success of occlusion was characterized as the reduction of cerebral blood flow (CBF) down to 10% of baseline, which was verified by a laser Doppler flowmeter (Moor Instruments, Devon, England). The ICA was occluded for 1.5 h, followed by the removal of the suture to allow reperfusion. Mice were injected with adjudin (50 mg/kg, DMSO stock dissolved in corn oil at a dilution of 1:10) or DMSO (DMSO dissolved in corn oil at a dilution of 1:10) intraperitoneally immediately after reperfusion. The second injection at the same dose was performed 5 hours after the first administration, and the third injection was given 48 h after reperfusion, also with the same dose. The sham group (n = 5) underwent the same procedure without suture insertion. All of the animals were sacrificed 72 h after reperfusion.

### Measurement of infarct volume

Mice from each group were sacrificed 3 d after reperfusion; brain tissue was immediately removed and frozen in pre-chilled isopentane. The tissue was then cut into a series of 20-μm-thick coronal sections from the beginning of the infarct area to the end, with the distance between adjacent sections of 200 μm. The entire set of brain sections was immersed in 0.1% cresyl violet for 30 min and then rinsed in distilled water for 10 min. The infarct area in each section was calculated using the ImageJ software by the following formula: contralateral hemisphere area (mm^2^) - ipsilateral undamaged area (mm^2^). Infarct volume between two adjacent sections was calculated by this formula:

$$1/3\times h\;\left(S1+S2+\sqrt{S1\;*\;S2}\right)\;,$$

where S1 and S2 are the infarct areas of the two sections, and h is the distance between them. The total infarct volume was calculated by the sum of all infarct volume from each pair of adjacent sections [15].

### Behavioral assessment

Neurological status was assessed by an investigator who was blind to the treatment regimen, based on the modified neurologic severity scores (mNSS) system in which mNSS is a composite of motor, reflex and balance tests. Total neurological score was calculated as the sum of scores on limb flexion (range: 0 to 3), walking gait (range: 0 to 3), beam balance (range: 0 to 6) and reflexes absence (range: 0 to 2). Therefore, neurologic function was graded on a scale of 0 to 14 (normal score 0; maximal deficit score 14) as previously described [16].

### Immunohistological staining

Brain cryosections (20 μm in thickness) were fixed with absolute methanol in a -20°C freezer for about 10 min, blocked with 10% normal donkey serum and incubated with one of the following primary antibodies: mouse anti-CD11b antibody (1:100 dilution, BD Biosciences, San Jose, CA, USA); rabbit anti-Occludin, rabbit anti-ZO-1, rabbit anti-IgG, rabbit anti-JAM-A, and rat anti-CD31 antibodies (all at 1:100 dilution, Life Technologies, CA, USA). After being washed with PBS, sections were incubated with Alexa-488-conjugated secondary antibody (1:200 dilution, Life Technologies), and nuclei were stained with 4,6-diamidino-2-phenylindole (DAPI) (1:500 dilution, Beyotime Institute of Biotechnology, China). Confocal microscopic images were acquired using a confocal laser-scanning microscope (Leica TCS SP5 II, Germany).

### Western blot analysis

Western blot analysis was performed as previously described with some modification [17]. The ischemic regions of left striatum and cortex were lysed in lysis buffer (Thermo Scientific, Rockford, IL, USA) containing 10 μM leupeptin and 200 μM phenylmethylsulfonyl fluoride. The lysates were centrifuged at 12,000 g for 20 min at 4°C, and the supernatants were collected. The protein concentration was measured using the BCA assay kit (Thermo Scientific). Total proteins (40 μg) were loaded on 6 to 10% SDS-polyacrylamide gel electrophoresis and were transferred to a nitrocellulose filter membrane (Whatman). The membranes were incubated with primary antibodies at 4°C overnight and then hybridized with appropriate HRP-conjugated secondary antibody (1:5000 dilution, Jackson) at room temperature for 1 h. After membranes were washed, the immunoreactive bands were detected by enhanced chemiluminescence (ECL) (Thermo Scientific), and images were captured by using the ChemiDoc XRS system (BioRad, Hercules, CA, USA). The primary antibodies used were as follows: Occludin/JAM-A/ZO-1 antibodies (1:500 dilution, Invitrogen) and β-actin (1:1000 dilution, Santa Cruz Biotechnology, CA). The intensity analysis was carried out using a Gel-Pro Analyzer (Media Cybernetics, Silver Spring, MD, USA).

### Enzyme-linked immunosorbent assay

The concentrations of TNF-α, IL-1β and IL-6 in sera from each group of animals were measured by platinum ELISA Kit: TNF-α (R&D Systems), IL-1β (R&D Systems) and IL-6 (R&D Systems), according to manufacturer’s instructions. The absorbance at 450 nm was determined using a microplate reader (Synergy2, BioTek). Protein concentrations were determined with a BCA Protein Assay Kit (Thermo Scientific). The TNF-α, IL-1β and IL-6 concentration in the serum was calculated with each standard and normalized against the protein of the samples.

### Real-time PCR

Total RNA was isolated from ipsilateral hemisphere of the adjudin group and the vehicle group and the counterpart of the sham group by using Trizol Reagent (Life Technologies, CA, USA) and was reverse-transcribed to cDNA using a PrimeScript RT reagent kit (TaKaRa). Quantitative real-time PCR was performed using SYBR Premix Ex Taq (TaKaRa) and the following primers: IL-6(sense 5′-tagtccttcctaccccaatttcc-3′ and anti-sense 5′-ttggtccttagccactccttc-3′); IL-1β (sense 5′-gcaactgttcctgaactcaact-3′ and anti-sense 5′-atcttttggggcgtcaact-3′); TNF-α (sense 5′-ccctcacactcagatcatcttct-3′ and anti-sense 5′-gctacgacgtgggctacag-3′); and Rplp0 (sense 5′-agattcgggatatgctgttggc-3′ and anti-sense 5′-tcgggtcctagaccagtgttc-3′). PCR was performed as previously described [13] at the following conditions: denaturing at 95°C for 10 s, followed by 40 cycles of 95°C for 5 s and 60°C for 30 s. Data were analyzed by using the comparative threshold cycle (Ct) method, and results were expressed as fold difference.

### Zymography

Activity of MMP-9 were tested by zymography as described previously [18] with slight modification. Tissue samples were prepared as for western blot but without denaturing before electrophoresis. Samples were loaded in a zymography specific buffer (BioRad, Hercules, CA, USA). After electrophoresis, the gel was incubated in Buffer 1 (2.5% Triton X-100, 50 mM Tris-HCl, 5 mM CaCl_2_, pH 7.6) twice for 40 min each with shaking. Then the gel was incubated in Buffer 2 (50 mM Tris-HCl, 5 mM CaCl_2_, pH 7.6) twice for 20 min each with agitation. After being washed twice, the gel was transferred to Buffer 3 (50 mM Tris-HCl, 5 mM CaCl_2_, 0.02% Brij-35, pH7.6) and incubated for 42 h at 37°C. Then, the gel was placed in staining solution (0.05% Coomassie Brilliant Blue, 30% methanol, 10% acetic acid) for 3 h, followed by washing in destaining solutions A (30% methanol and 10% acetic acid in double distilled water), B (20% methanol and 10% acetic acid in double distilled water), and C (10% methanol and 5% acetic acid in double distilled water) for 0.5, 1, and 2 h, respectively. Thereafter, the gel was photographed.

### Statistical analysis

All data are presented as mean ± SEM. Data were analyzed by a one-way ANOVA, followed by the Tukey post hoc test, with *P* values less than 0.05 considered statistically significant.

## Results

### Adjudin exerts a neuroprotective effect against ischemia/reperfusion injury

**Figure 1:**
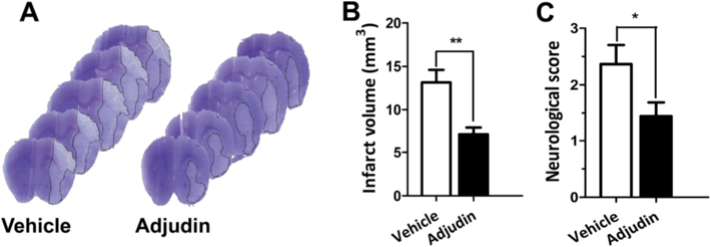
**Neuroprotective effect of adjudin on ischemia/reperfusion induced cerebral injury. (A)** Cresyl violet staining of brain sections from mice that underwent a transient middle cerebral artery occlusion (tMCAO) and were treated with either vehicle or adjudin 3 d after reperfusion. Dash line indicates infarct area. Quantification of the infarct volumes **(B)** and neurological scores **(C)** of adjudin-treated and vehicle-treated mice after tMCAO. Data were mean ± SEM, N = 4 to 5 in each group. *P <0.05.

In our previous work, administration of adjudin before surgery had no significant effect in reducing infarct volume from the pMCAO model [13]. However, the potential of this small molecule compound has not been fully tested. Therefore, in this study, we used the clinically more relevant tMCAO model. First, we aimed to test whether post-injection of adjudin could reduce infarct volume as well as neuroscore. In contrast with the vehicle group, mice treated with adjudin significantly reduced infarct volume by as much as 50% 3 days after reperfusion (Figure 1A,B, and Additional file 1: Figure S1). Moreover, adjudin treatment improved behavioral performance with the neuroscore plummeted by approximately 40% (Figure 1C). These findings illustrated that post-treatment with adjudin significantly attenuated ischemia/reperfusion induced cerebral injury.

### Adjudin attenuates microglial activation after ischemia/reperfusion

**Figure 2:**
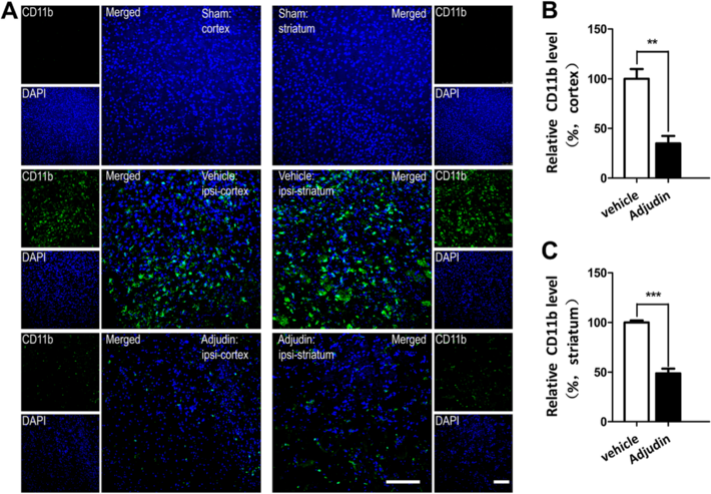
**Effect of adjudin on the activity of microglia after transient middle cerebral artery occlusion (tMCAO).** Immunofluorescence staining of CD11b in the cerebral cortex and striatum **(A)** from mice that underwent sham surgery (sham, top panel), tMCAO followed by vehicle treatment (vehicle, middle panel) and tMCAO followed by adjudin treatment (adjudin, bottom panel) 3 d after reperfusion. Scale bar = 100 μm. Microglial activation in the ischemic cerebral cortex **(B)** and striatum **(C)** is quantified by the intensity of CD11b immunofluorescence. Data were mean ± SEM, N = 4 in each group. **P <0.01, ***P <0.005.

We then investigated whether adjudin also affected microglia in the tMCAO model. CD11b signal, an indicator of active microglia, was revealed by fluorescence microscopy (Figure 2A). In the sham group, no obvious activation of microglia was expected, and no CD11b signal was detected in either the cortex or the striatum (Figure 2A, top panel). In the vehicle group, strong staining of CD11b as widely found in the two brain regions of the ipsilateral hemisphere (Figure 2A, middle panel). Contrarily, intraperitoneal administration of adjudin after reperfusion significantly inhibited the activation of microglia both in the cortex and the striatum where much less CD11b signal was detected (Figure 2A, bottom panel). Statistical analysis of the CD11b signal from brain sections of mice indicated that adjudin treatment significantly attenuated microglial activation in both brain regions (Figure 2B,C).

### Adjudin reduces ischemia/reperfusion induced cytokine production

**Figure 3:**
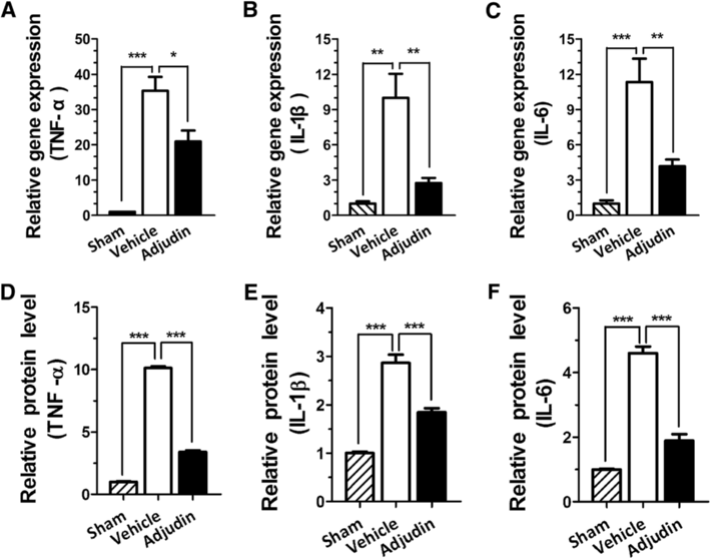
**Adjudin reduced ischemia/reperfusion induced cytokine production.** Bar graph shows the mRNA levels of TNF-α **(A)**, IL-1β **(B)** and IL-6 **(C)** in brain tissue 3 d after reperfusion. Data were expressed as average copies per copy of Rplp0, and normalized to sham group, from five separate experiments. Relative protein levels of TNF-α **(D)**, IL-1β **(E)** and IL-6 **(F)** in blood samples were measured with ELISA. Data were normalized against the protein level of the sham group. Data were presented as mean **±** SEM, n = 5 per group. *P <0.05, **P <0.01, ***P <0.005.

Ischemia/reperfusion-induced cerebral microglia activation could upregulate cytokine production. As adjudin could suppress microglia activation, we further explored if cytokine production in tMCAO models could be reduced after the administration of adjudin. Indeed, adjudin largely reduced transcription of TNF-α, IL1-β and IL-6 at mRNA levels (Figure 3A,B and C). Correspondingly, a significant decrease of protein levels of TNF-α, IL1-β and IL-6 had also been detected in the adjudin group (Figure 3D,E and F). In sum, these results illustrate that adjudin is a potent suppressor of ischemia/reperfusion-induced neuroinflammation. We also evaluated the iNOS expression at an earlier time point (1 d after reperfusion). There was a marked increase in the protein levels of iNOS, while adjudin treatment significantly reduced iNOS levels (Additional file 2: Figure S2). It is likely that adjudin could also inhibit the production of free radicals early after ischemia.

### Adjudin protects against ischemia/reperfusion-induced blood-brain barrier destruction

**Figure 4:**
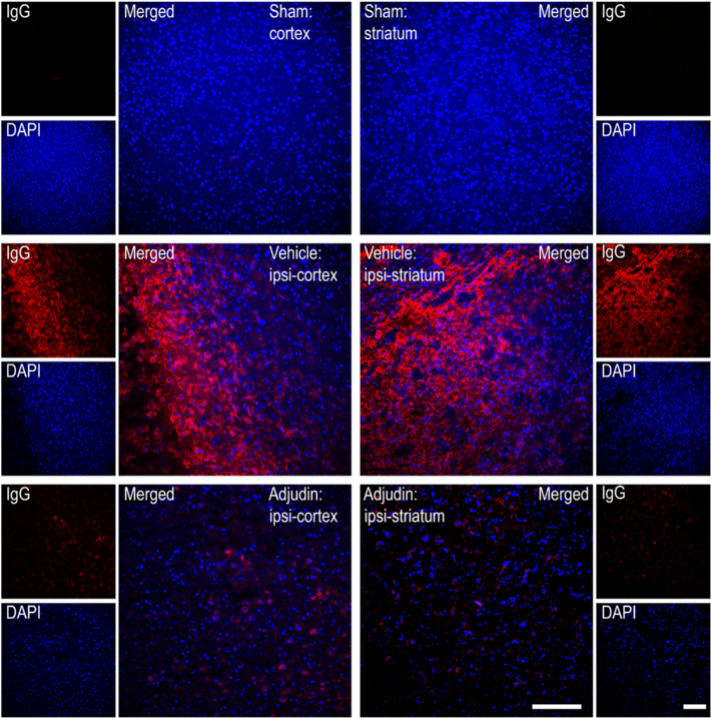
**Adjudin inhibited ischemia/reperfusion induced blood-brain barrier destruction assayed by detection of IgG.** Immunofluorescence staining for IgG (red) in the cerebral cortex and striatum from mice that underwent sham surgery (sham, top panel), transient middle cerebral artery occlusion (tMCAO) followed by vehicle treatment (vehicle, middle panel) and tMCAO followed by adjudin treatment (adjudin, bottom panel) 3 d after reperfusion, with DAPI staining for contrast. Merged images were shown at larger magnification, and scale bar =100 μm.

**Figure 5:**
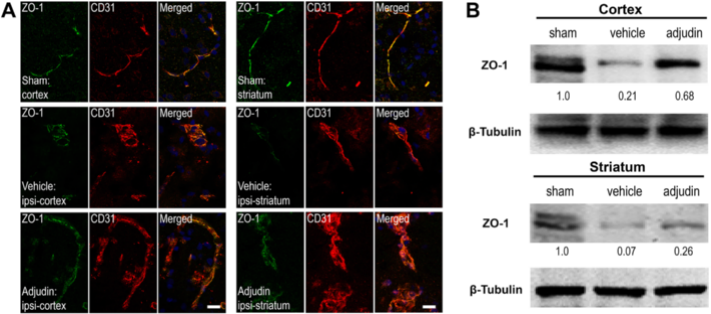
**Adjudin inhibited ischemia/reperfusion induced blood-brain barrier destruction assayed by tight junction related ZO-1. (A)** Immunofluorescence staining for ZO-1 (green) and CD31 (red) in the cerebral cortex and striatum from mice that underwent sham surgery (sham, top panel), transient middle cerebral artery occlusion (tMCAO) followed by vehicle treatment (vehicle, middle panel) and tMCAO followed by adjudin treatment (adjudin, bottom panel) 3 d after reperfusion. Merged images of ZO-1 and CD31 staining were also shown. Scale bar = 100 μm. **(B)** Representative western blot for ZO-1 levels in the cerebral cortex and striatum from mice of the sham, vehicle and adjudin groups. Densitometric value of the protein bands normalized to the respective β-tubulin was also shown.

**Figure 6:**
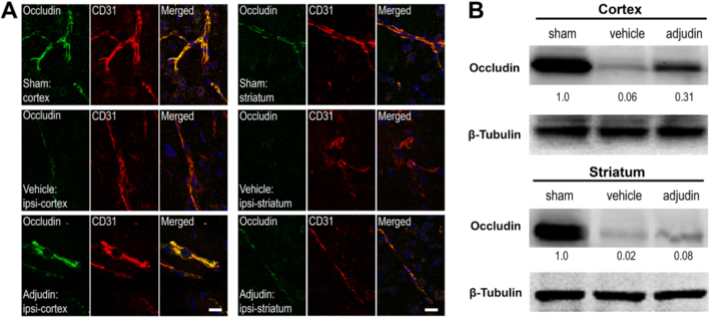
**Adjudin inhibited ischemia/reperfusion induced blood-brain barrier destruction assayed by tight junction protein Occludin. (A)** Immunofluorescence staining for Occludin (green) and CD31 (red) in the cerebral cortex and striatum from mice that underwent sham surgery (sham, top panel), transient middle cerebral artery occlusion (tMCAO) followed by vehicle treatment (vehicle, middle panel) and tMCAO followed by adjudin treatment (adjudin, bottom panel) 3 d after reperfusion. Merged images of Occludin and CD31 staining were also shown. Scale bar = 100 μm. **(B)** Representative western blot for Occludin levels in the cerebral cortex and striatum from mice of the sham, vehicle and adjudin groups. Densitometric value of the protein bands normalized to the respective β-tubulin was also shown.

**Figure 7:**
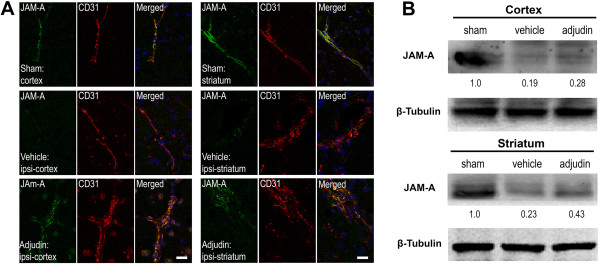
**Adjudin inhibited ischemia/reperfusion induced blood-brain barrier destruction assayed by tight junction protein JAM-A. (A)** Immunofluorescence staining for JAM-A (green) and CD31 (red) in the cerebral cortex and striatum from mice that underwent sham surgery (sham, top panel), transient middle cerebral artery occlusion (tMCAO) followed by vehicle treatment (vehicle, middle panel) and tMCAO followed by adjudin treatment (adjudin, bottom panel) 3 d after reperfusion. Merged images of JAM-A and CD31 staining were also shown. Scale bar = 100 μm. **(B)** Representative western blot for JAM-A levels in the cerebral cortex and striatum from mice of the sham, vehicle and adjudin groups. Densitometric value of the protein bands normalized to the respective β-tubulin was also shown.

Inflammation caused by ischemia/reperfusion in brain injury is often accompanied with BBB breakdown. Ruined BBB integrity increases leakage of serum proteins like IgG or albumin, which results in focal tissue hypoxia [19]. To further explore whether adjudin has a positive effect on the BBB destruction, we tested the integrity of BBB with immunofluorescence staining and western blot. A tremendous amount of IgG, which permeated both the cortex and striatum of the ipsilateral hemisphere of the vehicle group, was detected (Figure 4, middle panel), while mice treated with adjudin significantly reduced IgG leakage (Figure 4, bottom panel). Note that in the sham group, no IgG signal was found in the same brain regions (Figure 4, top panel), indicating an intact BBB was in place. To corroborate this result, tight junction (TJ)-related proteins ZO-1, Occludin, and JAM-A were examined by immunofluorescence microscopy in conjunction with CD31, an endothelial marker that also locates the BBB, and by western blot to determine the changes of protein levels. In the sham group, ZO-1 and CD31 signals were aligned most perfectly in both cortex and striatum (Figure 5A, top panel), where in the ipsilateral cortex and striatum of the vehicle group, such alignment became disorganized with ZO-1 signal being greatly reduced, indicative of damaged BBB (Figure 5A, middle panel). However, in the adjudin group, a certain degree of rescue of such superimposed lining of ZO-1 and CD31 was observed, suggesting that the BBB destruction after ischemia/reperfusion was attenuated (Figure 5A, bottom panel). Importantly, western blot analysis of lysates from the two brain regions also proved that the significant reduction of ZO-1 levels after ischemia/reperfusion (vehicle versus sham) could be rescued by adjudin treatment (Figure 5B). Similarly, we examined Occludin and JAM-A, two TJ component proteins, by immunofluorescence and western blot (Figures 6 and 7). Adjudin treatment obviously protected against the TJ protein reduction after ischemia/reperfusion injury, as indicated by the changes in the intensity of fluorescence signal and immunoblotting signal of Occludin and JAM-A from the sham, vehicle and adjudin groups (Figures 6 and 7). Together, these results further demonstrated that the BBB destruction after ischemia/reperfusion injury could be effectively rescued by adjudin treatment, possibly as a result of the attenuated neuroinflammation.

### Adjudin reduces ischemia/reperfusion-induced MMP-9 activity

**Figure 8:**
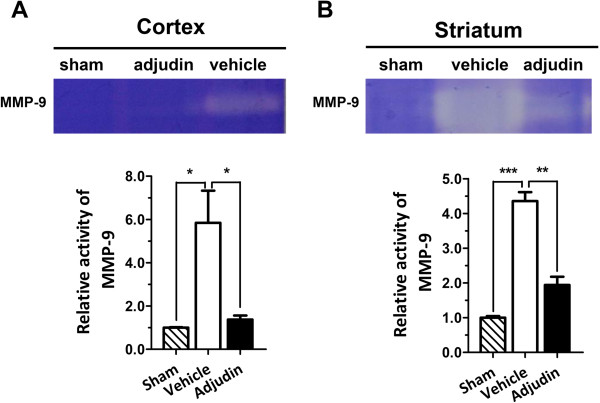
**Adjudin inhibited ischemia/reperfusion induced MMP-9 activation.** MMP-9 activity in the cerebral cortex **(A)** and striatum **(B)** from mice of the sham, vehicle and adjudin groups 3 d after reperfusion was assayed by zymography. Bar graphs summarized data from five groups and were presented as mean **±** SEM. *P <0.05, **P <0.01, ***P <0.005.

It is well known that matrix metalloproteinases such as MMP-9 could degrade the TJ proteins of the BBB. We continued to test the activity of MMP-9 in both the cortex and striatum from the sham, vehicle and adjudin groups by zymography. Lysates from these brain regions were processed and analyzed, and the zymograph clearly indicated that the upregulation of MMP-9 activity was significantly inhibited by adjudin treatment (Figure 8). This result partially explained that the protection of the BBB by adjudin treatment was mediated by an inhibition of MMP-9 activation.

## Discussion

In this work, we revealed that adjudin could decrease infarct volume and improve behavioral outcome in an ischemia/reperfusion-induced cerebral injury mouse model. Such neuroprotective effect was mediated by an inhibition of neuroinflammation as marked by a reduction of cytokine production, along with improved BBB integrity and suppressed MMP-9 activity. Together we presented strong evidence that adjudin, as an anti-inflammatory molecule, could be an effective neuroprotective agent to reduce ischemia/reperfusion injury.

In this study inhibition of pro-inflammatory cytokines could be mediated by both brain and systemic inflammation, which should not be separately considered. Inflammation caused by ischemia/reperfusion in the brain has been well studied, but systemic inflammatory responses after stroke have been characterized relatively less. Cerebral ischemia initiates a complex cascade of events that lead to focal brain injury, and inflammation plays an important role in those events. Inflammatory products derived from the ischemic area could cross the disrupted BBB and cause reciprocal systemic immune response as previous researches have reported. Cytokines like TNF-α and IL-6 are significantly increased in the peripheral immune system in both clinical and experimental stroke [20-22], and the activation of the peripheral immune system could also affect the already disrupted ischemic area, which could form a vicious circle and further spread the infarct area.

As our previous study has shown, adjudin could inhibit microglial activation in the pMCAO model [13]. In this research, a tMCAO model was introduced that could better mimic the clinical situation of stroke patients. With the withdrawal of the filament after 1.5 h occlusion, reperfusion of blood could induce another wave of damage to the penumbra area around the infarct core, mediated by ROS and inflammation [23]. CD11b staining revealed that post-treatment with adjudin also inhibited microglial activation in the tMCAO model. Concomitantly the heightened expression of cytokines TNF-α, IL-1β and IL-6 was also significantly attenuated by adjudin, further demonstrating diminished neuroinflammation by this agent.

As a cardiac-cerebral vascular disease, stroke is often companied with BBB disruption [24]; thus, maintenance of BBB integrity is a key strategy to protect brain from ischemia/reperfusion induced injury [15,25,26]. The BBB is a highly specialized structure located in the brain endothelial cells [27,28]. In cooperation with pericytes, astrocytes and microglia, the BBB could prevent plasma components such as leukocytes from infiltrating the brain [29]. In many neurodegenerative diseases like multiple sclerosis and Alzheimer’s disease, BBB integrity is compromised, leading to the leakage of blood cells, which further aggravate inflammation and generate neurotoxic products that can finally result in neuron death [30-32]. In this study, IgG immunofluorescence staining showed a decreased infiltration of this molecule in the adjudin-treated group versus the vehicle group, reflective of a better preserved BBB. Immunofluorescence staining and western blot analysis of ZO-1, Occludin and JAM-A further demonstrated that adjudin preserved BBB integrity in ischemia/reperfusion injury models.

TJ is the core part of the BBB, which is located in the tightly sealed monolayer of brain endothelial cells (BEC) [29]. With complex molecular interaction, TJ confers BBB the capacity to preclude blood substance from permeating [33]. In many experimental neuronal disease models TJ-related proteins like ZO-1, Occludin and JAM-A proved to be reduced, which consequently compromised the integrity of the BBB [34]. MMPs, which were significantly increased after ischemia/reperfusion-induced brain injury, were considered to be responsible for reducing ZO-1 and Occludin [35,36]. Numerous prior studies indicated that activated microglia could release MMPs after stroke [8]. Hence a ‘vicious circle’ may form: BBB disruption caused by the ischemia/reperfusion facilitates infiltration of macrophages and other immune cells in the blood, which aggravates the inflammation and further destroys the integrity of the BBB. As a consequence, the core infarction area spreads towards the peripheral area and brings severe damage that cannot be reversed. Our data indicated that adjudin could break this ‘circle’ by inhibiting the activation of microglia. Consequently, further escalation of brain injury could be attenuated.

Even though the activity of MMP-9 could be significantly inhibited by administration of adjudin, the underlying mechanisms still remain largely unrevealed. NF-κB could be the upstream regulator of MMP-9, and activity of MMP-9 could be suppressed by the inhibition of the NF-κB pathway [37,38]. In our previous work, we have already demonstrated that adjudin could inhibit NF-κB activity *in vitro*. But whether NF-κB still plays a role in the pathway in which adjudin exerts its neuroprotective effect *in vivo* needs to be studied. Mechanisms underlying BBB preservation by adjudin is worthy of further research. Adjudin is known not to perturb the blood-testis barrier even when administered at a much higher concentration; it only disrupts the adhesion between the sperm and the supporting cells [10]. Adjudin’s capacity to preserve the barrier function in the brain after injury, on the other hand, offers an intriguing point to the studies in reproductive physiology.

## Abbreviations

ACA: anterior cerebral artery; BBB: blood-brain barrier; BEC: brain endothelial cells; CBF: cerebral blood flow; CCA: common carotid artery; DAPI: 4:6-diamidino-2-phenylindole; ECA: external carotid artery; ECL: enhanced chemiluminescence; ELISA: enzyme-linked immunosorbent assay; ICA: internal carotid artery; IL: interleukin; mNSS: modified neurologic severity scores system; LPS: lipopolysaccharide; NO: nitric oxide; NSAID: non-steroidal anti-inflammatory drugs; PBS: phosphate-buffered saline; PCR: polymerase chain reaction; pMCAO: permanent middle cerebral artery occlusion; tMCAO: transient middle cerebral artery occlusion; TJ: tight junction.

## Competing interests

The authors declare that they have no competing interests.

## Authors’ contributions

WX conceived the project, coordinated the study, analyzed the data and drafted the manuscript. TL designed experiments; performed animal experimental procedures, behavioral assessment, ELISA, Real-time PCR, immunohistological staining, western blots; analyzed the data; and drafted the manuscript. TZ conducted Zymography analysis, immunohistological staining and western blot. HY participated in the animal experimental procedures, and conducted part of the behavioral assessment and data analysis. HS assisted in the experimental design and data analysis. All authors read and approved the final manuscript.

## Supplementary Material

Additional file 1: Figure S1 Assessment of relative microglia activation and lesion size after adjudin treatment. **(A)** Quantification of immunostaining signals of CD11b in the ipsilateral hemisphere, which was pooled from serial sections of four animals in each group. Results were normalized to the vehicle group (100%). **(B)** Relative infarct volume changes after adjudin treatment, which was a replot of Figure 1B. Click here for file

Additional file 2: Figure S2 Adjudin inhibited ischemia/reperfusion induced iNOS expression after 24 h reperfusion. Representative western blot for iNOS levels in the cerebral cortex and striatum from mice of the sham, vehicle and adjudin groups. Densitometric value of the protein bands normalized to the respective β-tubulin was also shown. Click here for file
