# Supplementary figures and images for: Adjudin protects against cerebral ischemia reperfusion injury by inhibition of neuroinflammation and blood-brain barrier disruption
Source: J Neuroinflammation. 2014 Jun 14;11:107. doi: 10.1186/1742-2094-11-107 (PMC4132223; doi:10.1186/1742-2094-11-107)

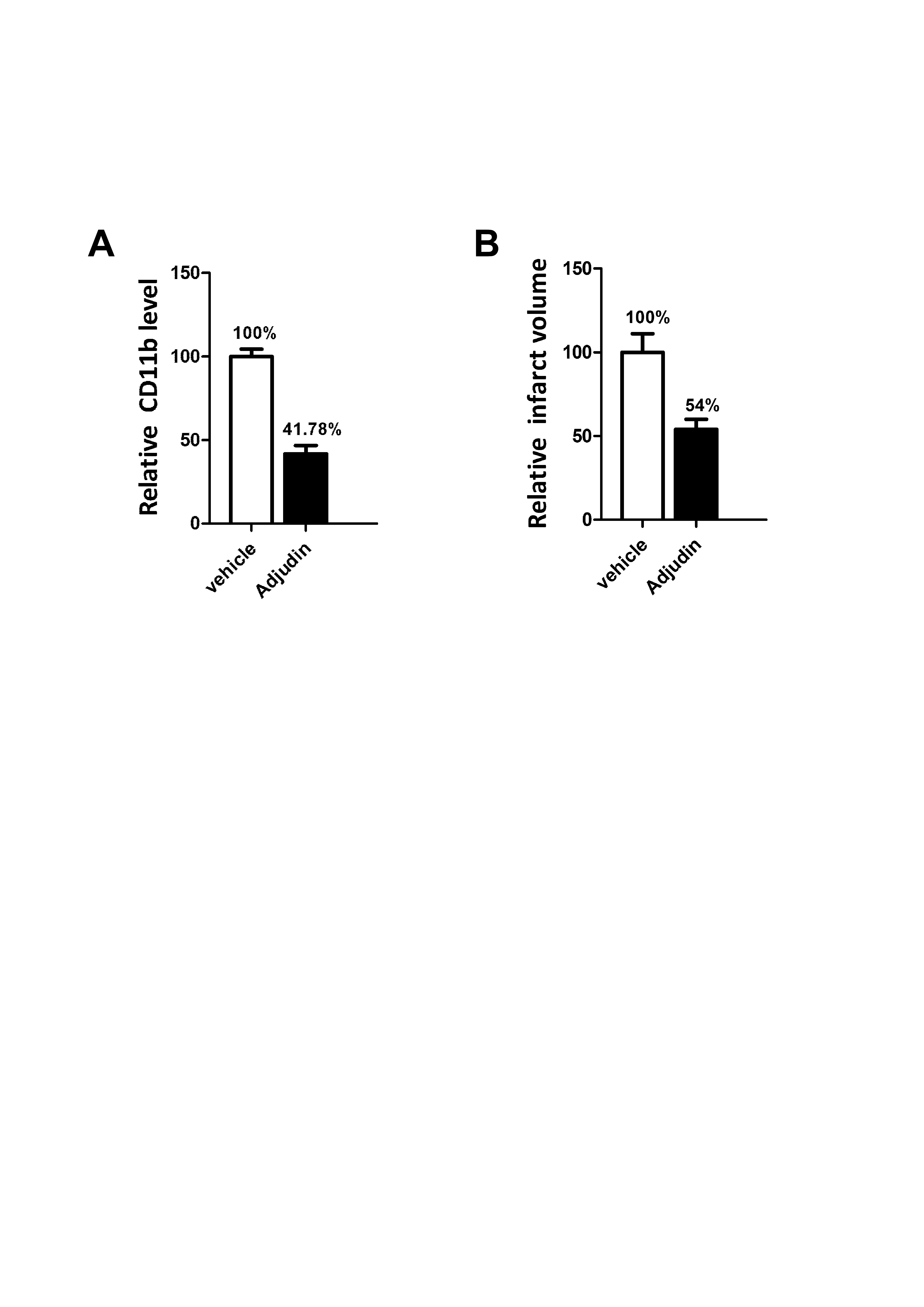

Supplement: Additional file 1: Figure S1 — Assessment of relative microglia activation and lesion size after adjudin treatment. (A) Quantification of immunostaining signals of CD11b in the ipsilateral hemisphere, which was pooled from serial sections of four animals in each group. Results were normalized to the vehicle group (100%). (B) Relative infarct volume changes after adjudin treatment, which was a replot of Figure 1B. [file 1742-2094-11-107-S1.jpeg]

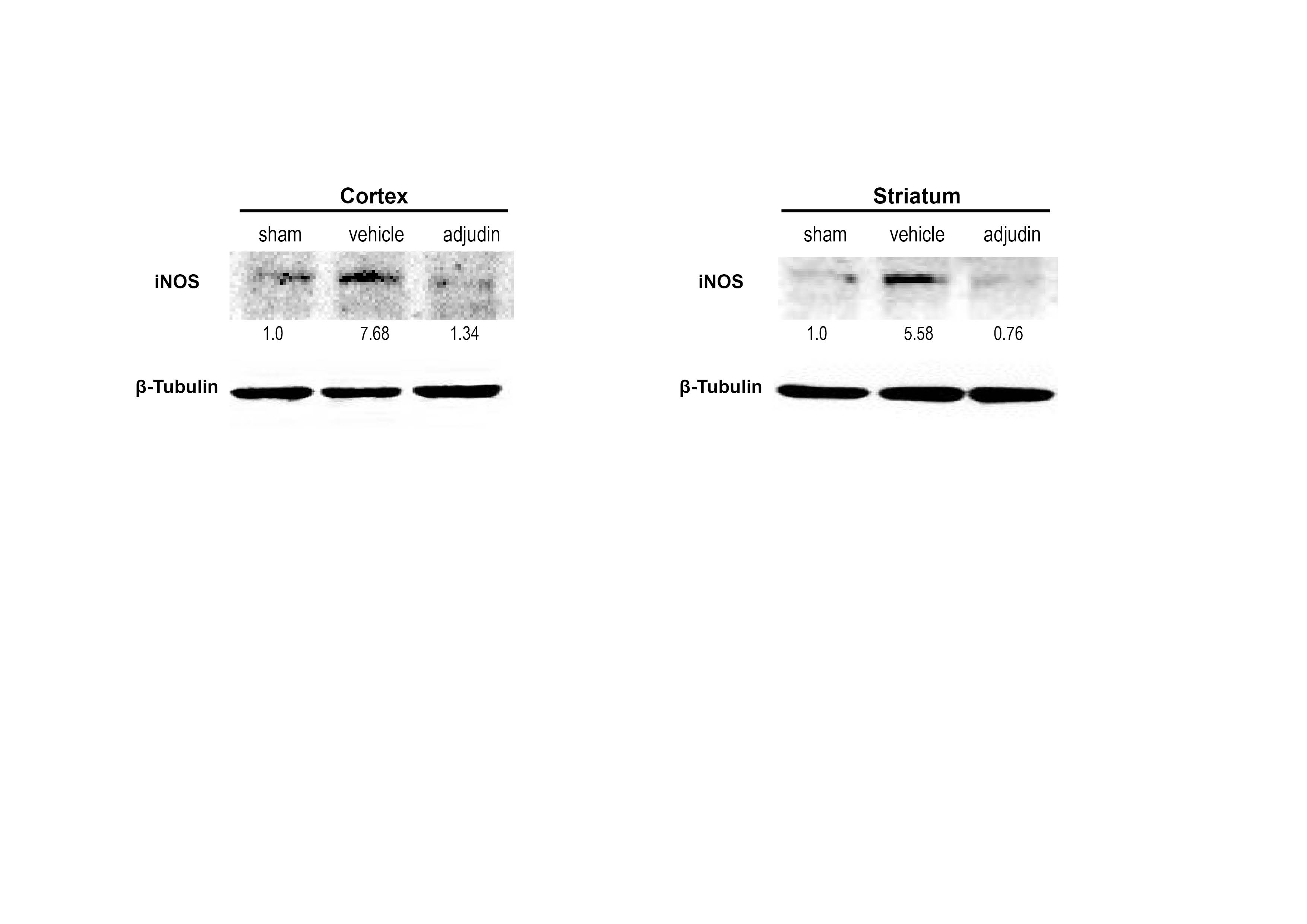

Supplement: Additional file 2: Figure S2 — Adjudin inhibited ischemia/reperfusion induced iNOS expression after 24 h reperfusion. Representative western blot for iNOS levels in the cerebral cortex and striatum from mice of the sham, vehicle and adjudin groups. Densitometric value of the protein bands normalized to the respective β-tubulin was also shown. [file 1742-2094-11-107-S2.jpeg]
